# Enhancing Documentation Standards of Complications in Informed Consent for Neck of Femur Fracture Surgery

**DOI:** 10.7759/cureus.96220

**Published:** 2025-11-06

**Authors:** Aaisha Shahbaz, Rohma Shahbaz, Yash Dinesh, Fahad Imami, Rizwan Akbar, Deepa Bose

**Affiliations:** 1 Trauma and Orthopaedic Surgery, University Hospitals Birmingham NHS Foundation Trust, Birmingham, GBR; 2 Trauma and Orthopaedic Surgery, Combined Military Hospital Multan, Multan, PAK; 3 Trauma and Orthopaedic Surgery, College of Medicine and Health, University of Birmingham, Birmingham, GBR; 4 Surgery, Pakistan Air Force Hospital, Islamabad, PAK; 5 Trauma and Orthopaedics, Hayatabad Medical Complex Peshawar, Peshawar, PAK

**Keywords:** clinical audit, neck of femur fractures, orthopedic surgery, postoperative complication, surgical informed consent

## Abstract

Background

Neck of femur (NOF) fractures are a major cause of morbidity in the United Kingdom. Operative management is the standard of care, and informed consent is essential to ensure patients understand potential benefits and complications arising during the surgical management of NOF fractures. This study aimed to assess and enhance the quality of Consent Form 1 documentation of complications of NOF fracture surgery as per the British Orthopaedic Association (BOA) and to assess improvements following focused interventions.

Methodology

A closed-loop audit was conducted in the Trauma and Orthopaedic Department, Heartlands Hospital, Birmingham, from February to October 2024. In the first cycle, we analyzed the Consent Form 1 retrospectively for the documentation of complications. Interventions for improvement were performed, including teaching sessions and posters on consent documentation. The second cycle assessed post-intervention practice. The study encompassed patients with capacity who underwent fixation, hemiarthroplasty, or total hip replacement (THR). Documentation was considered complete if it included all common, less common, and rare complications.

Results

The initial documentation rates were modest, with fewer than 50% of forms recording pain, catheterization, avascular necrosis, hip stiffness, limb length discrepancy, and mortality. Post-intervention improvements were observed, including pain, avascular necrosis, and death for NOF fixation, alongside better values of infection, altered wound healing, pulmonary embolism, and bone damage for hemiarthroplasty. For THR, improvements were seen in the documentation of bleeding, dislocation, blood clots, prosthetic wear, infections, altered wound healing, bone damage, and death.

Conclusions

Targeted teaching and visual reminders improved documentation, but there are persistent gaps. Further strategies are required to standardize consent practices and ensure complete patient information in line with BOA guidance.

## Introduction

Neck of femur (NOF) fractures are among the most common fragility fractures in the United Kingdom, with 65,000 cases reported each year [1]. They are classified as intracapsular or extracapsular based on their relationship to the hip capsule [2]. As per the National Institute for Health and Care Excellence Clinical Guidelines 124, the recommended management for NOF fractures is operative management on the day of or the day after admission [3], as early hip surgery within 48 hours is associated with lower mortality risk and decreased rate of complications [4,5].

Informed consent before any intervention is integral to current medical practice, as it ensures that patients are made aware of the benefits, risks, and alternatives of treatment, while empowering them to make decisions about their care. As per guidance from the General Medical Council, patients must be informed of the risks and complications associated with treatment, regardless of rarity [6]. As such, careful documentation of these discussions is essential for both patient safety and medico-legal protection.

Previous UK-based studies have highlighted limitations in orthopedic consent. An audit conducted by Bajada et al. revealed that consent forms for orthopedic procedures often lacked comprehensive information about potential complications [7]. Additionally, Bakhiet et al. described gaps in documenting complications in consent forms [8]. These studies demonstrate that, despite the existence of established guidelines, variability in practice persists in hospitals across the United Kingdom.

To overcome the discrepancies in orthopedic consent forms, the British Orthopaedic Association (BOA) has endorsed the use of consent forms available on orthoconsent.com and considered it to be the standard of consent practice [9]. Building on this context, this audit was undertaken to assess whether the consent forms for the surgical intervention of NOF fractures at Birmingham Heartlands Hospital adequately documented all possible complications of surgical management of NOF fractures. We assessed the documentation of complications for different surgeries performed as the treatment for NOF fractures, i.e., NOF fracture fixation with dynamic hip screw or cephalomedullary nail for extracapsular NOF fractures and hip hemiarthroplasty or total hip replacement (THR) for intracapsular NOF fractures. This audit aimed to determine the extent to which the Consent Form 1 for Neck of Femur Fracture Surgical Intervention contained the complete documentation of complications according to BOA-endorsed guidelines. The objective of this audit was to enhance the documentation of common, less common, and rare complications occurring in the surgical management of NOF fractures.

## Materials and methods

This was a closed-loop clinical audit comprising two audit cycles in which the quality of consent form documentation of NOF fracture surgery was assessed. This audit was conducted in the Department of Trauma and Orthopaedic Surgery of Birmingham Heartlands Hospital, University Hospitals Birmingham. The first cycle was conducted from February to April 2024. Intervention was implemented in June and July 2024, followed by a second cycle from August to October 2024.

The gold standard for consent documentation was derived from BOA-endorsed guidelines on orthoconsent.com. Documentation was considered complete if it included all the common (2% to 5%), less common (1% to 2%), and rare complications (<1%), as mentioned in the guidelines.

Retrospective data collection was performed during both cycles of the audit. All patients who had the capacity to sign Consent Form 1 and underwent NOF fracture fixation, i.e., dynamic hip screw or cephalomedullary nail insertion, hemiarthroplasty, or THR, during the study period were included in the study. Patients who had signed Consent Form 4 were excluded. Two trauma and orthopedic surgical registrars performed the data collection from the Department of Medical Records by reviewing the consent form using a structured proforma. Before the data collection, both registrars underwent training on predefined standards mentioned in the data collection proforma to ensure consistent application of the criteria. Variables that were recorded included date of surgery, type of surgery, and the complications written on the consent form.

Following the first cycle, the results were presented in the departmental audit meeting, and interventions were performed. The interventions included teaching sessions on adequate consent practices and the display of posters highlighting the complications that need to be documented on the forms.

Data analysis was conducted using SPSS version 25 (IBM Corp., Armonk, NY, USA). Common, less common, and rare complications, being categorical variables, were analyzed as percentages, and the results from the first and second cycles were compared descriptively to assess for improvement after the intervention. Additionally, the differences between the first and second cycle were analyzed using the chi-square test or Fisher’s exact test, as appropriate. The Fisher’s exact test was used when the expected frequency in any cell was less than five. A p-value <0.05 was considered statistically significant.

This project was registered with the local clinical audit department under the CARMS Audit Code 21332. As it was conducted as part of a service evaluation, formal ethical approval was not required. Patient confidentiality was maintained throughout, and no identifiable data were collected.

## Results

In the first cycle of the audit, 127 patients underwent NOF surgery, of whom 84 had Consent Form 1 and were included in the study. Among the 84 patients, 35 (41.7%) underwent fixation, 39 (46.4%) hemiarthroplasty, and 10 (11.9%) THR. In the second cycle, there were a total of 145 patients who had surgical management for NOF fractures, of whom 94 were included as they had signed Consent Form 1. Among these, 51 (54.3%) underwent NOF fixation surgery, 25 (26.6%) hemiarthroplasty, and 18 (19.1%) THR.

The results of the first cycle showed that for NOF fixation surgery, including dynamic hip screw and cephalomedullary nail fixation, there was less than 50% documentation rate for postoperative pain, need for catheterization, limb length discrepancy, avascular necrosis, hip stiffness, and death. In the documentation of patients who underwent hip hemiarthroplasty, pain, altered wound healing, and death had a less than 50% documentation rate. For THR, pain, wearing and loosening of the implant, altered wound healing, bone damage, and death had a less than 50% documentation rate.

In the second cycle, improvement was seen in the documentation of pain, increasing from being mentioned in six (17%) consent forms to 17 (33%) consent forms, bleeding documentation increased from 28 (80%) to 43 (84%), infection from 31 (89%) to 47 (92%), catheterization from two (6%) to four (8%), altered leg length from four (11%) to eight (16%), and avascular necrosis from zero to eight (16%) for NOF fracture fixation surgery. However, the rate of documentation of blood clots dropped from 32 (91%) to 43 (84%), hip stiffness from eight (23%) to 10 (20%), nerve damage from 31 (89%) to 43 (84%), bone damage from 27 (77%) to 38 (75%), and blood vessel damage from 31 (89%) to 41 (80%). We found that the documentation of avascular necrosis improved significantly (p < 0.05).

For hip hemiarthroplasty, improvements were seen in the documentation of infection (34 (90%) to 24 (96%)), altered wound healing (seven (18%) to 10 (40%)), pulmonary embolism (31 (80%) to 22 (88%)), bone damage (22 (56%) to 21 (84%)), and blood vessel injury (29 (74%) to 19 (76%)). In the statistical analysis of documentation of complications of hip hemiarthroplasty, statistically significant improvement was found only in the documentation rates of bone damage (p < 0.05). For THR, improvements were seen in almost all categories except pain (four (40%) to four (22%)), limb length discrepancy (eight (80%) to 14 (78%)), pulmonary embolism (nine (90%) to 16 (89%)), nerve damage (10 (100%) to 13 (72%)), and blood vessel damage (nine (90%) to 14 (78%)). Despite the improvements in percentages for the rest of the complications, none were found to be statistically significant. The findings of both cycles are summarized in Tables 1-3.

**Table 1:** Summary of the documentation rates of the complications associated with neck of femur fracture fixation.

Complications of neck of femur fracture fixation	Consent forms with documented complications, n (%)		P-value	Difference (%)
	First cycle	Second cycle		
Pain	6 (17)	17 (33)	0.09	+16
Bleeding	28 (80)	43 (84)	0.605	+4
Blood clots	32 (91)	43 (84)	0.513	-7
Infection	31 (89)	47 (92)	0.710	+3
Urinary catheterization	2 (6)	4 (8)	1.000	+2
Altered leg length	4 (11)	8 (16)	0.754	+5
Avascular necrosis (for dynamic hip screw only)	0 (0)	8 (16)	0.019	+16
Hip stiffness	8 (23)	10 (20)	0.716	-3
Nerve damage	31 (89)	43 (84)	0.754	-5
Bone damage	27 (77)	38 (75)	0.780	-2
Blood vessel damage	31 (89)	41 (80)	0.313	-9
Death	7 (20)	10 (20)	0.964	0

**Table 2:** Summary of the documentation rates of the complications associated with hip hemiarthroplasty surgery.

Complications of hip hemiarthroplasty	Consent forms with documented complications, n (%)		P-value	Difference (%)
	First cycle	Second cycle		
Pain	18 (46)	9 (36)	0.422	-10
Bleeding	33 (85)	21 (84)	1.000	-1
Limb length discrepancy	30 (77)	16 (64)	0.262	-13
Joint dislocation	33 (85)	20 (80)	0.738	-5
Blood clots	34 (87)	22 (88)	1.000	+1
Infection	35 90)	24 (96)	0.640	+6
Altered wound healing	7 (18)	10 (40)	0.051	+22
Pulmonary embolism	31 (79)	22 (88)	0.505	+9
Nerve damage	32 (82)	19 (76)	0.751	-6
Bone damage	22 (56)	21 (84)	0.022	+28
Blood vessel damage	29 (74)	19 (76)	0.882	+2
Death	8 (21)	12 (16)	0.751	-5

**Table 3:** Summary of the documentation rates of the complications associated with total hip replacement surgery.

Complications of total hip replacement surgery	Consent forms with documented complications, n (%)		P-value	Difference (%)
	First cycle	Second cycle		
Pain	4 (40)	4 (22)	0.400	-18
Bleeding	7 (70)	14 (78)	0.674	+8
Limb length discrepancy	8 (80)	14 (78)	1.000	-2
Dislocation	8 (80)	17 (94)	0.284	+14
Blood clots	9 (90)	17 (94)	1.000	+4
Prosthesis wear/loosening	4 (40)	11 (61)	0.433	+21
Infection	9 (90)	18 (100)	0.357	+10
Altered wound healing	3 (30)	6 (33)	1.000	+3
Pulmonary embolism	9 (90)	16 (89)	1.000	-1
Nerve damage	10 (100)	13 (72)	0.128	-28
Bone damage	4 (40)	10 (56)	0.430	+16
Blood vessel damage	9 (90)	14 (78)	0.626	-12
Death	2 (20)	4 (22)	1.000	+2

## Discussion

This audit evaluated how thoroughly complications were recorded on Consent Form 1 for patients undergoing surgery for NOF fractures. The first cycle showed that many risks, particularly those considered less common or rare, were often missing from the documentation. Some improvement was seen after focused interventions such as educational sessions and posters, especially in documenting general surgical risks. However, significant gaps remained, with several procedure-specific complications still seldom recorded. These findings reflect a wider issue seen in orthopedic practice and echo results published in several similar audits.

Studies conducted in other hospitals have reported a strikingly similar pattern. Perera et al. observed that general complications such as bleeding, infection, and thromboembolic disease were usually included in consent forms, whereas orthopedic-specific issues such as prosthetic failure, altered leg length, or avascular necrosis were hardly ever recorded [10]. Our audit displayed the same imbalance: common risks were typically documented, while more domain-specific complications were often neglected. Shah et al. reached comparable findings, with mortality, implant dislocation, and leg length discrepancy omissions being given the spotlight [11]. The Oldham audit recorded just over half of the recommended complications on average before intervention, though compliance improved considerably once consent stickers and teaching were introduced [12]. Davey et al., who looked more broadly at orthopedic trauma consent, showed that even basic elements such as site and side were not always present on forms before systematic learning was provided [13]. Together, these studies underline that incomplete documentation is not unique to our department but rather a persistent problem across the specialty.

The gradual progress seen after our intervention is encouraging, especially the higher rates of recording infection, bleeding, and pain. They show that simple, inexpensive measures can have an effect when awareness is raised. However, the fact that rare complications such as neurovascular injury, hip stiffness, or death remained absent from most forms suggests that education alone is insufficient. To ensure that everything is covered, structural help may be needed, such as pre-filled consent forms or standard checklists. Evidence from other centers shows that these methods can raise compliance levels far higher than just teaching sessions.

The clinician’s experience and seniority in gaining consent are also likely to influence quality. In many hospitals, including ours, junior personnel frequently complete consent forms for NOF surgery. These clinicians are under pressure in emergency settings and may not always remember the full range of potential consequences. Perera et al. noted that junior trainees recorded fewer specific risks compared with senior colleagues, and it is likely that this dynamic affected our own results [10]. Another consideration is that the absence of a risk from the written form does not prove that it was never mentioned to the patient. Discussions may encompass more than what is documented. Even so, both ethically and legally, accurate documentation is crucial; the written record should reflect the conversation that has taken place.

Our audit has evident strengths. It used a closed-loop design, which allowed us to monitor the effect of interventions. It was benchmarked against nationally recognized recommendations from orthoconsent.com. The inclusion of patients undergoing fixation, hemiarthroplasty, and THR provided breadth and enabled comparisons across procedures. However, some limitations must be acknowledged. The cycles relied on retrospective data collection, which raises the possibility of missing or incomplete forms. Additionally, observer bias could exist, although the data collection was performed by individuals who were trained in the orthoconsent.com complications checklist before data collection. Patients requiring Consent Form 4 were excluded, and therefore, practices in those without capacity, which is a large subgroup in the NOF patients, remain unknown. We have mainly reported descriptive outcomes, and the project evaluated documentation only, not patient understanding or recall of risks. It is also unclear whether the improvements will be sustained, especially given the frequent turnover of junior employees.

The implications for practice are clear. Standardized consent forms or stickers that follow the BOA principles would encourage doctors to list all pertinent risks and lessen reliance on recollection. Incorporating consent education into junior doctor induction and providing refresher sessions during the year would help strengthen expectations. Posters and other visual cues should be maintained in clinical areas to further reiterate complications that are commonly missed. Patients could benefit from additional written or digital material to help guide clinicians through the procedure and ensure that they are fully informed of the risks. Finally, regular re-auditing should be done in departmental governance, creating a continuous improvement cycle for further compliance monitoring and sustaining progress.

## Conclusions

This audit confirmed that documentation of complications for NOF surgery remains incomplete, particularly for less common and rare risks. Educational interventions led to modest improvements, but significant shortfalls persisted. These findings are in line with previously published work and highlight the need for a combination of education, structural changes, and cultural reinforcement. Thorough documentation is essential for informed consent, promoting patient autonomy, fostering confidence, and guaranteeing medico-legal protection in addition to being a matter of regulatory compliance. Given the frailty and vulnerability of the patient population undergoing surgery for hip fractures, it cannot be stressed enough how crucial a strong and trustworthy consent form is. To integrate best practices into orthopedic trauma care, persistent efforts backed by systemic solutions are necessary.
